# The Cambrian fossil *Pikaia*, and the origin of chordate somites

**DOI:** 10.1186/s13227-024-00222-6

**Published:** 2024-02-01

**Authors:** Thurston Lacalli

**Affiliations:** Biology Department, University of Victoria, Victoria, V8W-3N5 Canada

**Keywords:** Somite innervation, Nerve cord origins, Ancestral chordates, Undulatory swimming, The enteropneust model

## Abstract

The Middle Cambrian fossil *Pikaia* has a regular series of vertical bands that, assuming chordate affinities, can be interpreted as septa positioned between serial myotomes. Whether *Pikaia* has a notochord and nerve cord is less certain, as the dorsal organ, which has no obvious counterpart in living chordates, is the only clearly defined axial structure extending the length of the body. Without a notochord to serve as a reference point, the location of the nerve cord is then conjectural, which begs the question of how a dorsal neural center devoted to somite innervation would first have arisen from a more diffuse ancestral plexus of intraepithelial nerves. This question is examined using hemichordates as a reference point, first for the information they provide on the organization of the ancestral deuterostome nervous system, and second, extending the analysis of E. E. Ruppert, to explain why neural infoldings like the enteropneust collar cord would first have evolved. Both implicate the medial surface of the anterior-most part of the metacoel as the likely site for the evolution of the first somites. The analysis highlights the importance of the somatobranchial condition in chordates, meaning the linkage between the anterior trunk, *hox1* expression, and the beginning of the gill series and somites. This feature is arguably a valid criterion by which to assess extinct taxa from the Cambrian that resemble chordates (e.g., vetulicolians and yunnanozoans), but may be unrelated to them. In a more speculative vein, the nature of the dorsal organ is discussed, including the possibility that it is an expanded neural tube combining neural and support functions in one structure.

## Background

**Fig. 1:**
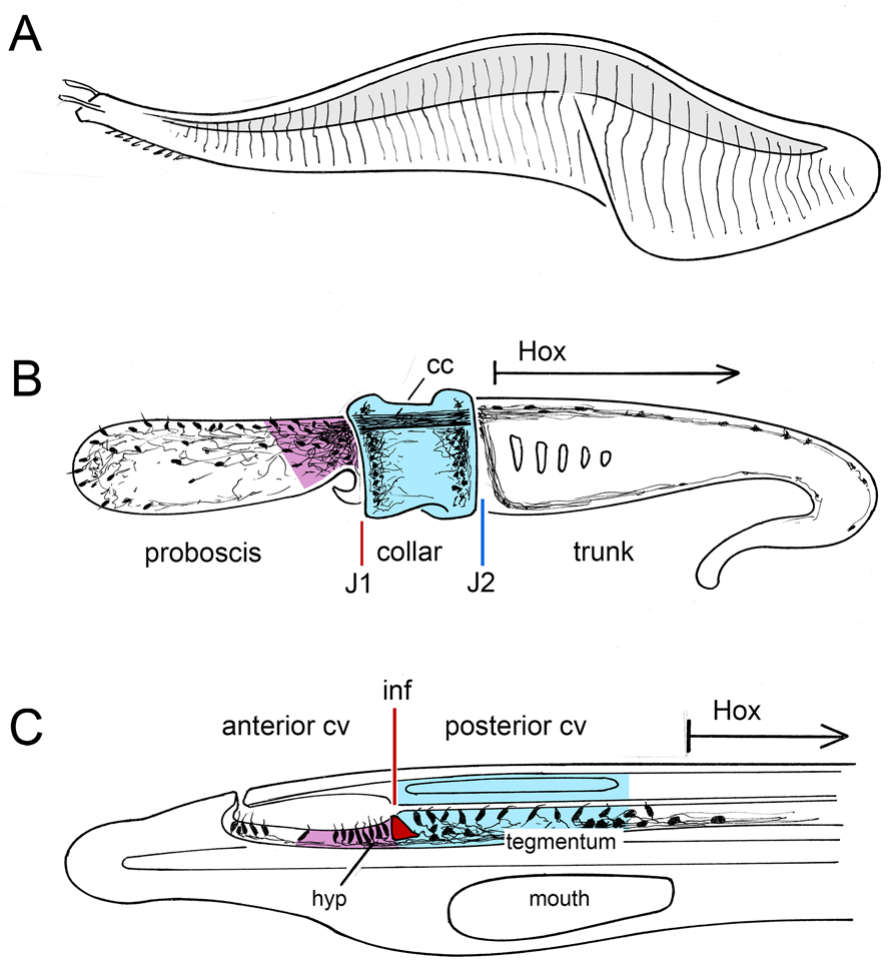
**A** *Pikaia gracilens*, from the Middle Cambrian Burgess Shale, a schematic drawing showing the main features discussed here: the small head and associated appendages, possibly gill-related, the series of putative somites, and the dorsal organ (shaded). The animal is portrayed with a pronounced bend in the body as if a wave of muscle contraction were propagating along it, but whether the body could flex in this way is a matter of conjecture (see [6] for a discussion). Figure 2 shows the head region in more detail. **B** An overview of the enteropneust nervous system, from [26], showing fibers from the extensive proboscis plexus projecting caudally through the collar cord (cc). The junctions (J1 and 2) between the three subdivisions of the body correspond in molecular terms with landmarks in the vertebrate brain, J1 with the *zona limitans* and J2 with the mid-hindbrain boundary [22], which means the neurogenic domain between these (in blue) is equivalent to the dien-mesencephalon of chordates as defined in molecular terms [12] while the neurons occupying the region immediately forward of J1 (in purple) correspond to types localized to the vertebrate hypothalamus. The trunk marks the beginning of the zone expressing Hox genes as shown. **C** The front of an amphioxus larva for comparison showing what is effectively the brain, i.e., the cerebral vesicle (cv), whose anterior and posterior parts map as shown to the vertebrate hypothalamus (hyp) and the basal dien-mesencephalon (tegmentum), which are colored to match their hemichordate counterparts in B. In amphioxus, the junction between the anterior and posterior cv occurs at the infundibular organ (red), which marks a major change in organization [28], implying very different evolutionary histories for the regions forward of this point and caudal to it. In contrast, the transition from the posterior cv to the rest of the nerve cord is more gradual, with Hox expression beginning at about the midpoint of somite 2

A number of Cambrian fossils from the exceptionally well-preserved Lagerstätten of the Burgess Shale and Chengjiang deposits have been interpreted as being basal chordates or related to them. Some, notably vetulicolians and yunnanozoans, remain problematic [1–4]. *Pikaia*, however, since its 2012 re-description by Conway Morris & Caron ([5], here CMC), and the analysis and critique of their findings shortly thereafter by Mallatt & Holland ([6], here MH), has been widely accepted as having clear chordate affinities. The body (Fig. 1A) bears a series of vertical striations resembling somite boundaries, which implies the presence of serial muscle blocks (i.e., myotomes), and is flattened and streamlined as might be expected of an animal that swam in an undulatory fashion. Waves of contraction, controlled by nerves, would presumably have been propagated along the body, a process that in living chordates depends on the presence of an axial nerve cord and a notochord that acts as a compression strut. However, while CMC provisionally identify axial traces that could correspond to these (their Figs. 12D,F, 13D, 14L,N, 15B-E,G, 16E), those attributed to the notochord are not as substantial as would be expected of a notochord modeled on modern chordate lines [6]. This is problematic, first, because the stiffness of rods and tubes increases with increasing radius, so the smaller the notochord the less it resists bending. But also, given how small the supposed notochord of *Pikaia* is in relation to the height of the myotome (see CMC’s Fig. 8D), a scaffolding of connective tissue as stiff as the notochord itself would have been required to link the latter with the somites in a functionally useful way. This begs the question of why a notochord would be needed at all when the basal lamina separating the putative myotomes is as sturdy as it appears to be in *Pikaia*, since a series of box-like chambers, given sufficient internal hydrostatic pressure, would support the body by themselves. Because the shape of the myotomes suggests that *Pikaia* did not engage in rapid escape swimming [7], it may not have required a particularly robust support system in any case, and there are, in addition, other ways to support the body during undulatory swimming (see Comments to [7]). Coupled with these objections is the fact that the best-preserved axial traces are ventral, identified by CMC as a possible blood vessel (their Figs. 12B, 13D, 15E-G), so a corresponding dorsal trace located close to the expected position of a notochord would be needed to complete the vascular circuit. In sum, the various axial traces preserved in *Pikaia* pose a number of difficulties of interpretation where CMC’s putative notochord, the “deep” notochord in MH’s terminology [6], is especially problematic. This is vexing because the notochord is so central to our concept of what a chordate is and how it functions [8].

Rather than dwelling on features *Pikaia* may not have, my intent with this account is to focus on those that it does, namely a body whose musculature can reasonably be supposed to be organized in repeating units comparable to chordate myotomes. I then want to consider how these would have originated. To function, the emergent myotomes would have required innervation, so I take the nervous system as my starting point, using enteropneusts and amphioxus as reference points. This leads to a consideration of the neuroanatomical innovations required to innervate deep structures, including emergent somites. Here I draw on the ideas of Ruppert [9] regarding the functional necessity of the collar cord, but interpreted in the context of our now much improved understanding, derived from gene expression data, of how the bodies and nervous systems of basal deuterostomes are patterned during development [10–12]. Based on the latter, there is an emerging appreciation of common features shared across the three deuterostome phyla despite morphological differences, of molecular patterning first and foremost, but also of neural organization and neuronal cell types. The organisms themselves can then be appreciated as functioning entities, as opposed to being treated simply as assemblages of character traits, an important difference when it comes to thinking critically about the relation between living taxa and fossils potentially related to them. This is particularly relevant to interpreting *Pikaia*. Superficially it looks fish-like, but placing it near the base of the chordate lineage implies that it is less fish-like than amphioxus, which is itself more like an enteropneust than a fish in some key respects, as discussed below. Recognizing this is important if we are to correctly interpret *Pikaia* and understand its place in the chordate story.

While much of this exercise is necessarily speculative, it is speculative in a narrow sense, in laying out a series of arguments about the ancestral condition and likely directions of evolutionary change. The nature of the dorsal organ is also discussed, a topic that is speculative in a different sense, given that there are multiple interpretations of what it might be, but no way to choose between them. It is useful nevertheless to show by example the importance of considering all the possibilities, as some would place *Pikaia* closer to the lineages to which extant chordates belong than others.

A complicating factor for any discussion of early chordate evolution is the issue of dorsoventral inversion. The scenario developed here for somite origins could unfold as proposed whether chordates are inverted or not, and so is agnostic on this point. However, the degree of homology between structures would be different. For example, absent inversion, similarities between the hemichordate collar cord and the chordate nerve cord [13, 14] could plausibly be argued to be due to homology at levels up to and including the anatomical one. In other words, both being dorsal, they are potentially homologous as structures. Chordate-specific changes in the expression patterns of genes associated with dorsoventral axis specification would then be better explained as a consequence of the evolution of the chordate trunk, and hence the organizer, a key chordate innovation [15]. With inversion, in contrast, there could be homology at a genetic and cellular level, e.g., in how the two structures are specified and constructed, but they would have separate origins and occupy different locations. My own opinion tends towards chordates being un-inverted, the molecular evidence favoring a conserved mechanism of nerve cord specification across taxa being somewhat less convincing than it was [16]. Further, inversion followed by mouth repositioning requires a reorganization of the chordate head and anterior nerve cord [17, 18], changes for which there is as yet no clear evidence.

### The ancestral deuterostome: molecular and neuroanatomical insights

Investigating the origin of characteristic chordate features requires an appreciation of the context in which these evolved, which means understanding in some detail how the body of ancestral deuterostomes was organized. We are materially aided in this by the results of investigations into the molecular events that specify different body regions along the anteroposterior axis, how these map across taxa and, in particular, the neuroanatomical correlates of those maps. The genes involved, Hox genes for the trunk and a more diverse group of genes from different families for the head, are highly conserved across bilaterians as a group and can be used as markers for subdomains within the nervous system [19]. Hemichordates have proven particularly informative [10, 20–22], as their simple morphology conceals a molecular and neuroanatomical landscape that is surprisingly complex and directly comparable with that of both chordates and echinoderms [11, 22, 23]. An apt analogy to my mind is the Rosetta Stone, where the meaning of a text repeated in three scripts could be only understood because one of them was already known. In the case of deuterostome body plan, hemichordates play the role of the known script in providing a model for understanding the other two taxa and their relation to each other.

The body of the common ancestor of all deuterostomes can be supposed, based on the archicoelomate hypothesis [24], to have been divided into three parts (in hemichordates, the proboscis, collar and trunk [25]) supported, in anteroposterior order, by paired proto- meso- and metacoels. Based on recent evidence [26, 27], the hemichordate nervous system, long known to be plexus-like and intraepithelial, has proven to be more complex and regionally differentiated than previously supposed. The proboscis is supplied with sensory neurons and a dense plexus, which contributes to the tracts of the collar cord, while separate populations of neurons are found in the collar itself, and along the dorsal and ventral nerves supplying the trunk (Fig. 1B). Superficially, amphioxus looks very little like an enteropneust, but its nervous system is organized in a remarkably similar way. The first subdomain of the amphioxus nerve cord is the anterior cerebral vesicle (the anterior cv, see Fig. 1C) which ends at the infundibular organ. Based on morphological and molecular markers [12, 28, 29], the anterior cv maps to the proboscis plexus in hemichordates, its vertebrate counterpart being the basal forebrain to the end of the prethalamus. This accords with molecular evidence [22] that the *zona limitans intrathalamica* is the vertebrate counterpart of the junction between the enteropneust proboscis and collar (J1 in Fig. 1B). The vertebrate hypothalamus and its amphioxus counterpart lie just forward of this point [30–32], matched in enteropneusts by a corresponding part of the proboscis plexus (purple in Fig. 1B) having similar organization and neuronal subtypes [26].

The region immediately caudal to the vertebrate *zona limitans*, along with its amphioxus counterpart, the posterior cv, combine components from the thalamus proper and the basal midbrain to form a single domain referred to, on the molecular evidence, as the dien-mesencephalic primordium (Di-Mes for short, see [12]). This region (blue in Fig. 1C) maps to the hemichordate collar (blue in Fig. 1B). From the available data, the distinction between the pre- and post-*zona* domains (the pre- and post-infundibular domains in amphioxus; proboscis and collar in enteropneusts) is thus an ancient one that probably reflects a functional difference, first pointed out by Merker [33]: between an anterior, hypothalamus-like domain, apical and preoral in origin, principally concerned with sensory integration and the selection of action paths, and a postoral midbrain/tegmental domain that implements those actions. The junction between the two domains is notable in amphioxus for being a major point of transition in nerve cord organization, from an open lumen without a floor plate to a narrowed slit with a floor plate [28]. This serves as a further, and in my view important link between amphioxus and enteropneusts where, for the latter, the comparable transition is between two neural domains that are also organized very differently, as an open plexus forward of the proboscis/collar junction, and a canal with a floor plate behind that junction [26].

The second junction in enteropneusts is between collar and trunk (J2 in Fig. 1B) which corresponds to the junction between the midbrain and hindbrain in vertebrates [22]. Comparison with amphioxus is hampered here by the absence of a clear anatomical transition or a specific molecular marker beyond the beginning of nested Hox expression, at the level of approximately the midpoint of somite 2. This marks the transition from what is effectively the head, which in hemichordates would be the proboscis plus collar, to the rest of the body, i.e., the trunk.

It is useful at this point to compare the division of the body and coelomic and nervous systems into three parts, which I will refer in its modern (i.e., molecular and neuroanatomical) formulation, as the enteropneust model, with the chimera hypothesis [34]. The term chimera refers here to evidence that the nervous system, across bilaterians, combines an apical component shared with marine larvae, represented by the apical sensory plate and associated secretory centers [28, 35, 36], with a blastoporal component innervating mesodermal derivatives generated at gastrulation and responsible for locomotory control. The enteropneust model introduces a refinement by highlighting the further subdivision of the blastoporal component into an anterior collar-related portion and a caudal trunk-related one. The former, together with the proboscis, forms what is effectively the “head” of the animal, whose neuromuscular component (derived from the mesosome) is, and probably originally was, less concerned with locomotion per se than with feeding-related activities. This would include operating the tentacles if one accepts pterobranchs or something similar to them as plausible models for ancestral deuterostomes [37–39]. The locomotory control component of the nervous system would then have begun only with the trunk proper, corresponding with the beginnings of Hox gene expression.

The enteropneust model thus pays as much if not more attention to the second junction (J2 in Fig. 1B) as to the first (J1), which directs attention to the beginning of the hindbrain in vertebrates, its amphioxus equivalent [12], and the margin of the ambulacra in echinoderms [23]. This same head/trunk distinction has proven to be important in understanding evolutionary patterns in marine invertebrate larvae, which are in many cases effectively swimming heads, the cells responsible for producing the trunk having been sequestered or otherwise delayed in their development [40–42]. In comparison with the head, the fate of the trunk is more variable across deuterostome taxa, being lost in echinoderms, retained but of limited locomotory utility in hemichordates, and transformed entirely in chordates with the evolution of the organizer and somites.

**Fig. 2:**
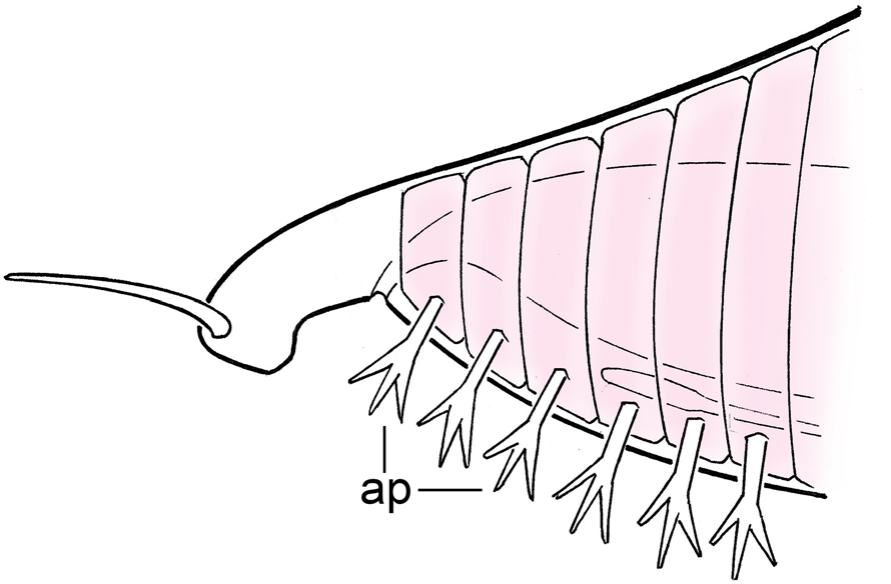
The anterior end of *Pikaia*, oriented with the mouth facing down, modified from Fig. 8C of [5]. The anterior appendages (ap) are generally interpreted as gills or gill-related structures, and repeat in register with the anterior segments (pink). The pharynx, dorsal organ and ventral blood vessel are shown in outline. The point of the diagram is to show that there is no evidence for appendages positioned forward of the putative somite series, so if the latter indicates the beginning of the trunk, the region constituting what is effectively the head of *Pikaia* is exceedingly small

The distinction between head and trunk can then be used to advantage in assessing fossils that otherwise resist taxonomic categorization. Examples would include both vetulicolians and yunnanozoans which, because they possess serial gill-like structures or openings, have been interpreted as both basal deuterostomes and chordates [1, 2, 6]. Their taxonomic position continues to be a matter of contention, the link with deuterostomes being, if anything, weaker than previously supposed [3, 43, 44]. My main concern in the present context is the positioning of the putative gills, in an anterior region clearly separate from the more trunk-like part of the body assumed to be responsible for locomotion. The term I would use for this condition is cephalobranchy (head gills) where, assuming the trunk can be defined by its locomotory function, places the gills anterior to this domain in a region that, based on the enteropneust model, and assuming these animals may be deuterostomes, must by default be head-like in character. This contrasts with the situation in living chordates, and also in *Pikaia* (Fig. 2), where the beginning of the gill series coincides with the beginning of the somite series, a condition I will refer to as somatobranchy (body gills) for want of a better term. By this criterion alone, *Pikaia*, even if it is not a chordate, would still have a valid claim to being a deuterostome. The regional subdivision of the body in vetulicolians and yunnanozoans, in contrast, though consistent with them being related to each other, makes it much harder to accept them as closely related either to extant deuterostomes or to *Pikaia*. There are alternatives, for example, if the anterior portion of the trunk in an ancestral vetulicolian-type chordate was first expanded to produce a region of replicated gill slits forward of the first somite that was later, in tunicates, enclosed to form the atrium. Conversely, vetulicolians and, by extension, yunnanozoans, could simply be tunicates with an everted atrium, but this would not explain their terminal anus and would make them at least as distant morphologically from ancestral chordates as other tunicates, and hence both specialized and less relevant to broader phylogenetic concerns.

### Somite origins: the link between neural invagination and deep structures

The nervous system of echinoderms and hemichordates consists mainly if not exclusively of intraepithelial tracts and plexuses in which synapses are rare or absent. Transmission is likely therefore to be largely by paracrine release, with the limitations on transmission speed and efficiency that implies. What is less appreciated is how closely this is mirrored in amphioxus, where a good fraction of transmission is also paracrine, but in addition, where synapses do occur, transmitter release occurs within the tracts themselves or, where the targets are mesodermal, across the basal lamina. In this respect it is the vertebrates that are atypical among deuterostomes in having nerves that penetrate the mesoderm to innervate effectors through direct synaptic contact. This is possible only because of early links formed between the nerve cord and differentiating neurons of neural crest origin that have migrated into the connective tissue, thereby establishing axonal pathways that can be used later to innervate mesodermal targets directly as needed [18]. The greater degree of flexibility this provides has arguably allowed vertebrates to reconfigure their musculoskeletal system across a wider range of body size than would otherwise be the case. Other deuterostomes are more restricted in this respect meaning, it would seem, that their body plan is less scalable.

**Fig. 3:**
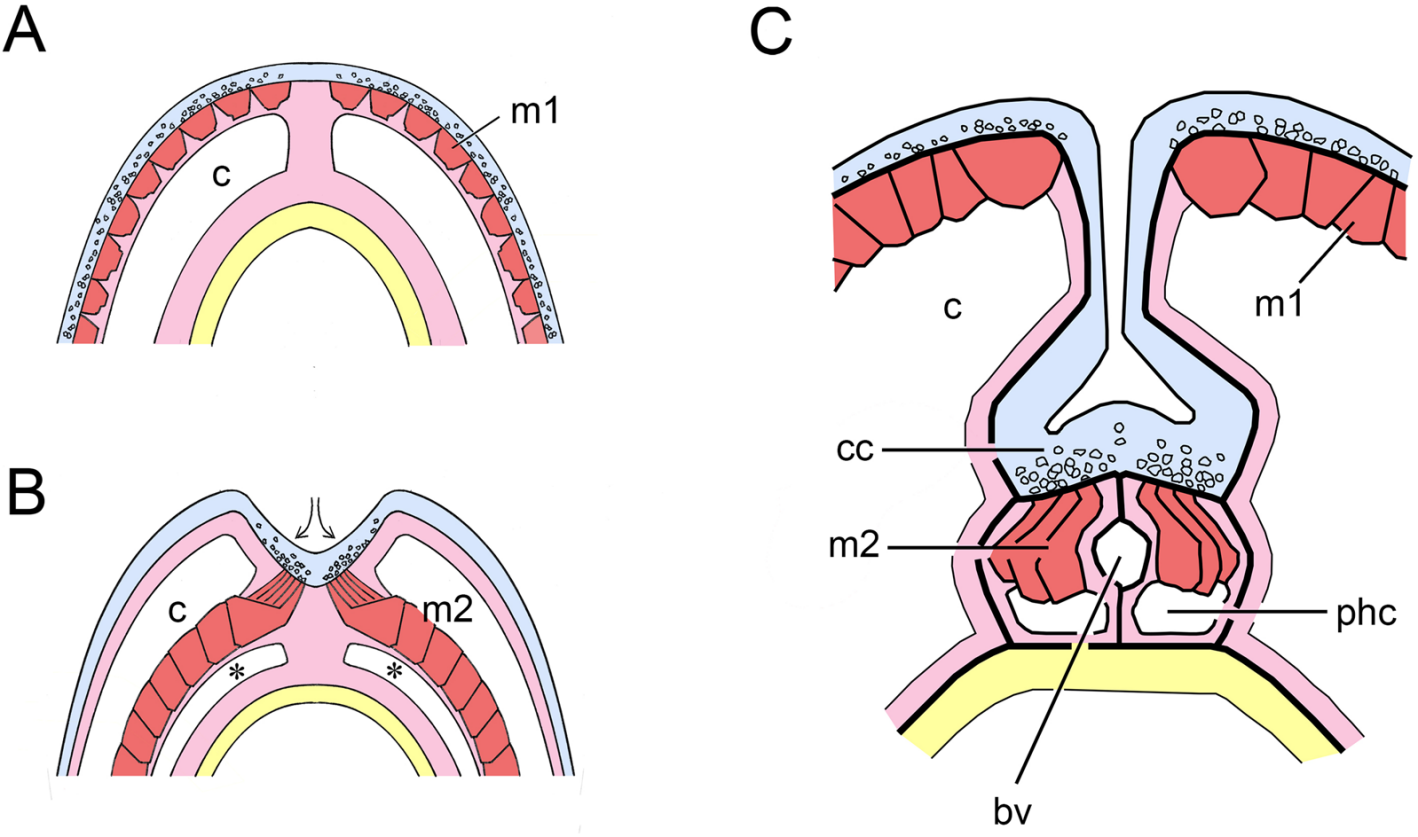
**A, B** Alternative ways to organize and innervate body musculature, modified from Ruppert’s Figs. 6–8 (see [9]). **A** represents a vermiform ancestral coelomate that moves (or burrows) by means of propagating waves of contraction that travel along the body. The muscles responsible (m1) are an intrinsic part of the outer mesothelium of the coelom (c), and are innervated by intraepithelial nerves (representative nerve fibers are shown in section as small open circles). **B** The situation in chordate somites, where the myotomal muscles (m2) lie along the inner surface of the coelom. To innervate these requires a fold or invagination of the neurogenic epithelium to bring the nerves (arrows) within contact range of the muscles. In effect this produces a rudimentary nerve cord that can then be shallow or deep as required. The region indicated by asterisks is a continuation of the coelomic cavity, as in chordates the muscles extend into the coelom in this way. My assumption is, that if *Pikaia* is a chordate, its musculature would necessarily be arranged in a similar fashion, along the inner surface of the coelom. Tissues are color-coded here and in subsequent figures: neural tissues and non-neural ectoderm in blue, mesoderm in pink (for epithelia and connective tissue) and red (for muscles), endoderm in yellow. **C** The specific example Ruppert uses to illustrate his point: the collar cord (cc) of an enteropneust, modified from Fig. 3 in [9]. The main coelomic cavity (c) belongs to the mesocoel, while the perihaemal coeloms (phc) are diverticula arising from the metacoel that project forward on either side of the medial blood vessel (bv). As explained in the text, the perihaemal coeloms are then ideally positioned to serve as models for the first somites

In the present context, the key point is the problem deuterostomes other than vertebrates face when it comes to innervating muscles that are distant from the body surface. The solution Ruppert highlights is to internalize the nerve plexus through the development of local infoldings of the neurogenic epithelium. There is both a general and a specific point to be made here. The general point (Fig. 3A, B) is that for muscles positioned elsewhere than on the outer face of the coelom, intraepithelial innervation is of little use without a means of bringing the nerves themselves closer to their targets. The specific point relates to the dorsal collar cord in enteropneusts (Fig. 3C). The muscles in this case are longitudinal fibers that develop from the walls of the perihaemal coelomic compartment adjacent to the dorsal blood vessel in the collar, which then aid in anchoring the proboscis at its base, acting in effect as a proboscis retractor muscle. The perihaemal coeloms develop, not from the mesocoel, but the metacoel. They then extend forward, through the mesocoel, in a fashion typical for coelomic diverticula employed for purposes of support in hemichordates. Comparing hemichordates with amphioxus, we find the latter faces much the same problem with its myotomes. The solution is, again, to have an invaginated cord in order to bring the innervation closer to its target, with the remaining distance bridged by cellular extensions from muscle fibers that project to the outer surface of the nerve cord [31].

A valid question then is whether the amphioxus nervous system, despite its chordate-type nerve cord, should really be considered more centralized than that of hemichordates [57, 58]. If we mean by “centralized” that a greater proportion of neurons are localized to an internalized nerve cord, then that would seem to be the case. But that is simply a matter of certain neuronal subtypes being less broadly deployed across the ectoderm. If instead we are concerned with the level of organization, i.e., intraepithelial versus something more complex, then arguably the nervous systems of hemichordates and amphioxus are in essence very similar. My own observations on larval neuroanatomy in amphioxus accords with this, as do those of Ruppert [45] on the adult, that where nerves appear to penetrate the basal lamina, this is not an indication that they penetrate the mesoderm, but instead is a secondary consequence of the mesoderm having grown around to enclose them in sheaths of connective tissue matrix. The dorsal nerves are an example, as their transit between the myotomes results from exactly this process. Whether the generalization applies to all the nerves in amphioxus, i.e., that they never truly penetrate the mesoderm, remains to be determined. The prospect that it might highlights what is potentially a serious constraint on developmental strategy for this organism, in that every target requiring innervation would have to receive it early in development when distances to the nearest neuroepithelial tracts are minimal. The circuitous routes followed by nerves in adult amphioxus is the result, a consequence of the massive growth of surrounding structures that occurs between embryogenesis and the adult stage. *Pikaia,* if a basal chordate, would presumably be similar in this respect and face the same set of constraints.

**Fig. 4:**
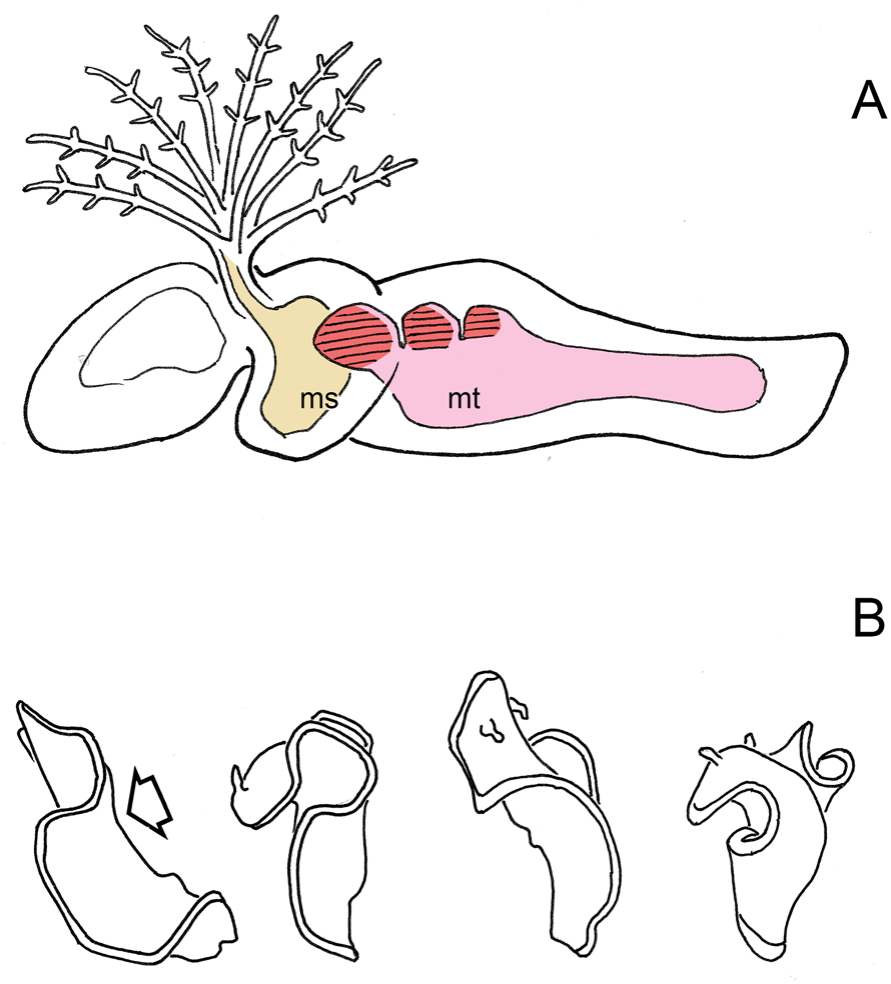
**A** How somites might first have originated, as diverticula from the anterior medial wall of the metacoel (mt), in a fashion similar to the formation of the perihaemal diverticula in Fig. 3C, which are then replicated in series. The animal is modeled on the Cambrian fossil *Gyaltsenglossus* [38], a supposed hemichordate that is enteropneust-like in its vermiform body, but also bears a crown of tentacles which, assuming they are homologs of pterobranch tentacles, would be supported as shown by the mesocoel (ms). Contractions of the three nascent somites (in red), which would be paired, would flex the body side to side. **B** Swimming by flexing: a swimming sequence for the Spanish Dancer, *Heterobranchus sanguineus*, traced at 1 s intervals. Arrow indicates the point of maximal dorsoventral flexure, but this motion also allows the expanded margins of the mantle to take advantage of water currents. Currents account for most of the translational motion observed in this sequence, as the flexures themselves serve mainly to alter body posture. Source: Wavelength Snorkeling Great Barrier Reef AVI, https://www.youtube.com/watch?v=V6H01cUSpfQ

To return to the collar cord and Fig. 3C: an invagination of the type shown could have evolved for various reasons and only been coopted secondarily for innervating muscles distance from the surface when those evolved. So, we may not have an explanation for why such an invagination would have evolved in the first instance, but there are clearly reasons to retain it once formed, and for it to be extended if the target muscles were subsequently to be replicated in series as somites are. Ruppert does not develop the argument in this direction, but the implications from his figures are clear, that this would explain how chordates came to innervate their somites by means of an invaginated nerve cord. The positioning of the hemichordate diverticula associated with the collar cord then becomes important, because they derive not from the mesocoel, but from the from metacoel, and so are trunk structures, as are chordate somites. And, if one pair of such diverticula can become two, and then three, and so on, the result would be as shown in Fig. 4A: a short series of closed or partially closed chambers along the medial surface of the anterior metacoel, each incorporating a block of longitudinal muscles. This would add a new functionality to the trunk, of lateral bending, without interfering with any creeping and/or burrowing activities it might otherwise be engaged in.

There is then a problem, that simple side-to-side flexures of the kind the first emerging somites would produce is not very effective as a means of locomotion. How then could it persist as the principal swimming mode through enough generations to be elaborated and refined into something as complex as a coordinated undulation? The answer is that there are animals today that survive quite happily while relying on similar body flexures to evade predators and to migrate over distance. Opisthobranch mollusks are a good example [46, 47], though their flexures are more often dorsoventral than side-to-side (Fig. 4B). This generates little in the way of forward movement, acting instead mainly to lift the animal off the substratum into the water column where it is then at the mercy of currents. The risk that this action attracts the attention of predators is mitigated in the case of opisthobranchs, including the Spanish dancer shown in the figure, by their being unpalatable and advertising the fact by their conspicuous coloration. It is reasonable then to ask whether predation would have been a problem for ancestral deuterostomes had they attempted this mode of locomotion, if, for example, an animal resembling *Gyaltsenglossus* in Fig. 4A took to flopping about in the water column. The answer is that, so long as this happened before the evolution of predators with eyes, being visually conspicuous is simply a non-issue. Even the most rudimentary method of locomotion would then have been a viable starting point for evolving a more efficient and well-controlled mode of locomotion.

As to the question of why chordate somites evolved in the first instance, my conjecture would be that it was dispersal rather than predator avoidance that provided the main impetus. For ancestral echinoderms and hemichordates, having a long-lived pelagic larval stage solves the problem of dispersal irrespective of how mobile the adult stage may or may not be [42]. For ancestral chordates, which may not have had a comparable pelagic larval stage [17], the burden of maintaining an adequate geographic range for the species would have fallen disproportionately on the adult, placing a premium on its ability either to swim or, at the very least, remain suspended in the water column long enough to be carried meaningful distances by currents.

### A more speculative issue: the dorsal organ

The best-preserved and most substantial axial structure in *Pikaia*, the dorsal organ, is among the most difficult to interpret in a chordate context [6]. It is an elongate ovoid extending along the length of the body, though attenuated anteriorly so as to make its full extent difficult to determine. A caveat is that the convention regarding which side of *Pikaia* is “up” could be mistaken, so the dorsal organ might in fact be ventral, perhaps part of the digestive tract. There is also the possibility that it is a buoyancy organ, so its position, either on the top or underside of the animal would depend on whether the specific gravity of its contents were less than or greater than that of sea water. Since my interest here is in options that make the dorsal organ part of the axial support system I will assume, with CMC, that it is located on the top of the animal as they illustrate it, and that this is the dorsal surface. For the dorsal organ then to provide meaningful support to the whole of the body, it would need to be attached to the myotomes via their basal laminae. Direct attachment would be limited to the dorsal part of each myotome, leaving the ventral part to be stiffened by other means, e.g., by the attachment between adjacent somites. How effective this would be is difficult to assess, but it is reasonable to suppose that undulations during swimming would be graded along the dorsoventral axis, with least amplitude at the stiffest point, i.e., dorsally, much like the folds in a curtain attached to a stiff rod. A more detailed analysis of the kind of motion such a system would produce requires knowing more than we do about the stiffness of each of the various elements, modes of attachment between them, and so on, leaving little more to be said. Instead, in this section, I will approach the question of the dorsal organ and its identity through a consideration of its tissue of origin.

**Fig. 5:**
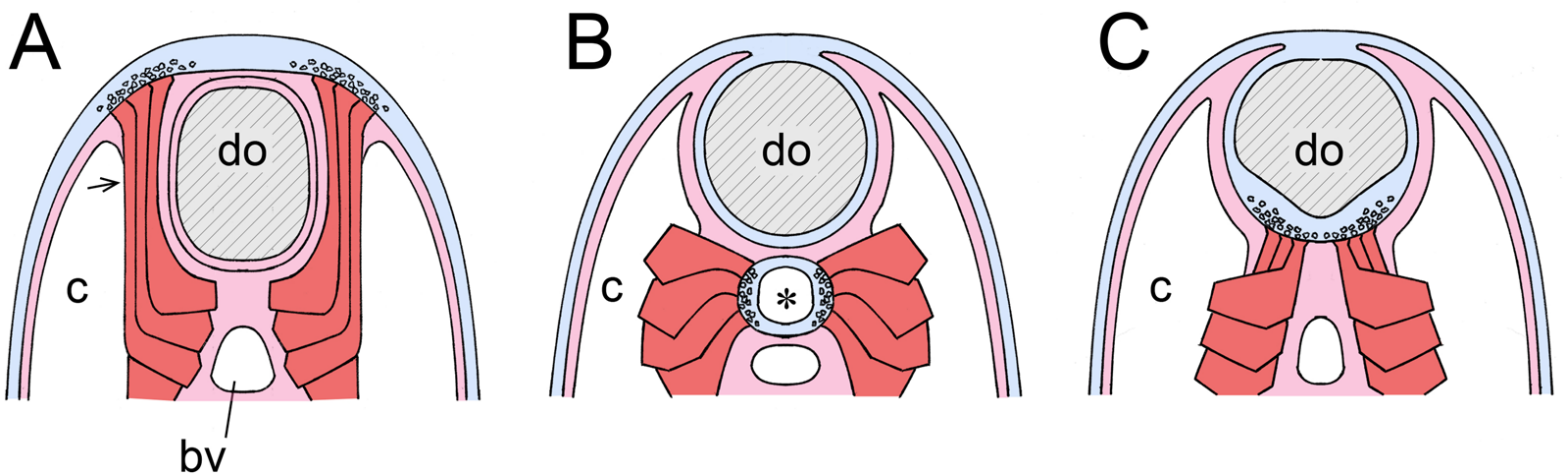
Three proposals for the source of the dorsal organ (do) and its relationship to other axial structures that *Pikaia* may possess. **A** Option 1: that it forms prior to neurulation, which may then not occur, and from mesoderm in the same fashion as the notochord, or it is a notochord, so that its contents, whether cells or secretions, would be mesodermal in origin. Any neural component would lie above it, so the only access deeper structures would have to innervation would be by means of muscle extensions (arrow), in the same way that neuromuscular connections are made in amphioxus. **B** Option 2: that it forms after neurulation as a secondary invagination positioned above the nerve cord (*). It is shown here (in blue) as ectodermal in origin, so its contents would be ectodermal secretions, or it might have a cuticular lining. A variant of this option would be to have neurulation followed by a dorsal expansion of the coelomic system to produce a similar chamber of mesodermal origin, so it would be pink in the figure, and the contents would be connective tissue and matrix. **C** Option 3: the dorsal organ is the neural tube, much expanded in size, which then innervates the somites while contributing to body support either because of its intrinsic structural strength, or because hydrostatic forces make it turgid, or a combination of the two. This is the least conventional of the three options but, as discussed in the text, there are reasons for giving it serious consideration

Assuming that the dorsal organ is not a digestive organ, it must be either ectodermal or mesodermal in origin. If the latter, then we may be looking at an enlarged notochord in an unusually dorsal position, probably filled with turgid vacuolate cells of a kind typical of both notochords and the hemichordate stomochord [48]. This would preclude there being a deeper, internalized neural tube, meaning somite innervation would be from nerves positioned above the dorsal organ, as shown in Fig. 5A. This is the least radical option and, if correct would place *Pikaia* firmly among chordates, as well as providing evidence that the notochord was more likely than not to have been a key component of the axial support system when chordate swimming first evolved. It accords also with the widespread view, shared by Ruppert [9], that the developmental link between the notochord and nerve cord in extant chordates argues for mutual dependence, so they would have evolved together.

The second option is that the dorsal organ is a tubular ectodermal derivative, perhaps lined and stiffened by cuticular material or some other kind of secretion. In this case, assuming the dorsal organ forms after neurulation, a nerve cord of typical kind would lie below it (Fig. 5B) possibly along with a notochord of a conventional kind (MH’s deep notochord, not shown in the figure, but which would then occupy the same location as the blood vessel that is shown). A variant on this second option, also not shown in the figure, is for the dorsal organ to be mesodermal rather than ectodermal, but produced, again, after neurulation. This could be accomplished by having parts of the coelom expand upward and around the nerve cord to form a separate tube above the latter, much as the fin boxes are formed in amphioxus. In that case, again, a deep notochord might or might not be present. This second option is then agnostic on whether the evolution of undulatory swimming depended on having a notochord or not.

The third option (Fig. 5C) is that the dorsal organ again arises from the ectoderm, but from neurogenic ectoderm, with the resulting tube being identical to the neural tube. It would admittedly be a rather peculiar neural tube, larger in relative terms than in modern chordates, and able to serve simultaneously as a structural support and conduit for nerve tracts. As to origins: even a simple neural infolding could potentially act to resist lateral bending and so begin the process by which a neural tube with a support function evolved. Adding a layer of cuticle or other molecular components with suitable physical properties would increase the stiffness of the initial fold and any tube later derived from it. Turning the fold into a closed tube adds a further possibility that the structure could then act as a hydrostatic organ, its turgidity modulated by suitably controlling internal solute concentrations and osmotic flows. Evidence in support of this third option should then be of two kinds: that there should be cuticular secretions or other secretory products specific to the neural tube, and that hydrostatic organs of some kind might be associated with it.

- (1) Secretions unique to the neural tube

The secretion of interest here is Reissner’s fiber, a complex glycoprotein secreted by the infundibular organ in amphioxus and in vertebrates by the floorplate and subcommissural organ, which is then transported along the length of the neural canal by ciliary action [49, 50]. Its function is not entirely understood, though a role in straightening the nerve cord during embryogenesis has now been documented [51]. The mechanism here appears to involve a sensory feedback circuit rather than a direct physical effect, but what is remarkable about Reissner’s fiber is that the basic component, the glycoprotein SCO-spondin, though present in other taxa [52], combines in chordates with other components to yield a product that rivals the secretions of mesodermal connective tissue in molecular size and complexity [53]. So there is the question of why the complexity, but also why this degree of complexity is specific to chordates. This may relate to role gene shuffling has played in chordate evolution [54], but the conjecture I would make is more one of functional necessity, on the assumption that the nerve cord may once have had a role in body support, and that SCO-spondin was necessary to this role, serving either to stiffen the cord or modulate osmotic effects.

- (2) Neural ducts with a role in water balance

On the assumption that a closed neural tube could act as a hydrostatic organ, there would need to be some means of controlling water balance. Possible candidate organs are the various neural ducts of tunicates, though it is difficult to know how much weight to give to poorly understood structures in so modified a group as tunicates. Tunicate neural ducts are typically funnel-shaped, ciliated, and connect to the front of the neural tube. Among the functions attributed to them is a role in ascidians maintaining water flow, first to the brain, and from there to the vascular system [55], while in salps there is evidence they modulate water flows directed into the developing brain [56]. Both functions are rather modest as contributions to the overall physiology of the animal, but they could represent surviving relicts of a past, more important function if the neural tube once had a role in body support. Both this and the previous point, relating to Reissner’s fiber, are hardly strong evidence that the dorsal organ is a nerve cord, but the possibility should not in my view be dismissed out of hand.

## Conclusions

If *Pikaia* is a chordate, then even without a notochord it would represent a comparatively advanced member of that lineage based on the way its body is organized and the mode of swimming that organization implies. *Pikaia* is nevertheless difficult to place in relation to living chordates. It has, on the one hand, structures with no obvious chordate counterpart (e.g., the dorsal organ and the apparent head shield, or “anterior unit” [6]), while on the other, the evidence for structures it should have, like the notochord and nerve cord, is equivocal. There are nevertheless questions concerning chordate origins that one can address using *Pikaia* as a point of reference, with that of somite origins being the most obvious. The problem, in essence, is how to convert a large coelom spanning the trunk to a repeating series of smaller coelomic compartments. Ruppert’s ideas on the hemichordate collar cord provide a useful starting point by highlighting the role of structures of this type in innervating muscles distant from the body surface. The extension of an initial, anterior invagination, to form an extended neural tube, would follow as the targets of innervation, the myotomes, were themselves replicated as an extended anteroposterior series. In this scenario, the neural tube can be seen as an innovation at the anatomical level that solves a histological problem, of the inherent limitations of intraepithelial innervation. Any chordate with somites would then be expected to have a neural invagination comparable to the collar cord that extends as far along the anteroposterior axis as the somites themselves, or a neural tube of comparable length. The argument for homology between the collar cord and the chordate nerve cord remains open, but the case in favor is supported by evidence at the cellular and molecular level of how similar the nervous systems of hemichordates and protochordates are, with vertebrates being, in some respects, an outlier.

The position of the neural tube in ancestral chordates would have depended on whether a notochord was present or not, which then begs the question of how the body was supported in the absence of a notochord. Since improvements to the locomotory abilities and efficiency of ancestral chordates can be assumed to have been progressive, it is by no means clear that a structural support as substantial as a notochord would have been required in the first instance to act as a compression strut. Whether *Pikaia* has a notochord or supports its body by other means remains an unresolved problem. If the dorsal organ, whatever its tissue of origin, is in some way concerned with body support, then *Pikaia* is potentially representative of a transitional step in the sequence by which chordates evolved body support systems of increasing efficiency. And, if the dorsal organ is not simply an overlarge notochord, one would have to conclude that a notochord of typical chordate type (MH’s deep notochord) evolved considerably later than the somite series. This would place the dorsal organ at center stage as an early but temporary solution to the problem of body support. An alternative, that *Pikaia* represents an independent, now extinct chordate lineage with somites but no notochord, is also possible, or it could once have had a notochord that has been lost as its functions were taken over by the later evolution of the dorsal organ. There remain, in sum, a number of unresolved questions concerning this remarkable fossil and how it is to be interpreted.

## Data Availability

Not applicable.
